# Supplementary material for: Molecular Interactions of Tannic Acid with Proteins Associated with SARS-CoV-2 Infectivity
Source: Int J Mol Sci. 2022 Feb 27;23(5):2643. doi: 10.3390/ijms23052643 (PMC8910432; doi:10.3390/ijms23052643)
Supplement: Supplementary file 1 [file ijms-23-02643-s001.zip › ijms-1594547-supplementary.pdf]

International Journal of *Molecular Sciences*

*Article*

# Molecular Interactions of Tannic Acid with Proteins Associated with SARS-CoV-2 Infectivity

**Mohamed Haddad [1,2,†], Roger Gaudreault [3,†], Gabriel Sasseville [3], Phuong Trang Nguyen [4], Hannah Wiebe [5], Theo Van De Ven [5], Steve Bourgault [4], Normand Mousseau [3] and Charles Ramassamy [1,2,*]**

## SUPPLEMENTARY INFORMATION

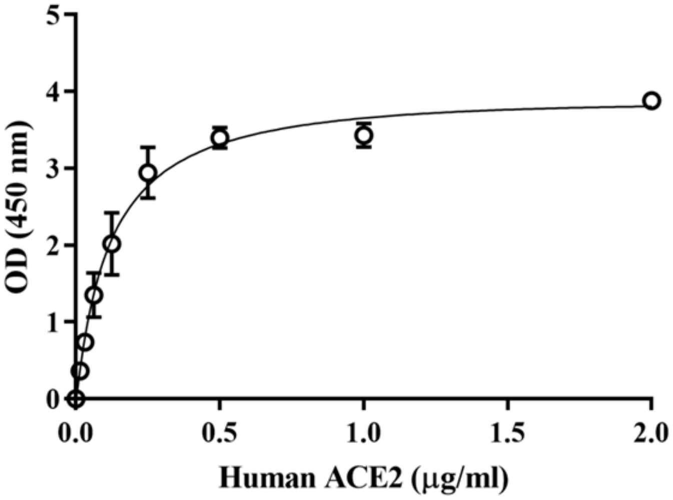


**Figure S1.** Concentration response curve for human angiotensin-converting enzyme 2 (ACE2) binding to immobilized severe acute respiratory syndrome coronavirus 2 (SARS-CoV-2) spike protein receptor binding domain (RBD) N501Y (0.5 μg/mL) using an increasing dose of human ACE2 protein (0.015 to 2 μg/mL). Results (mean ± standard error) are expressed as optical density (OD450) measurements using the Synergy HT multi-mode microplate reader with a 450 nm filter.

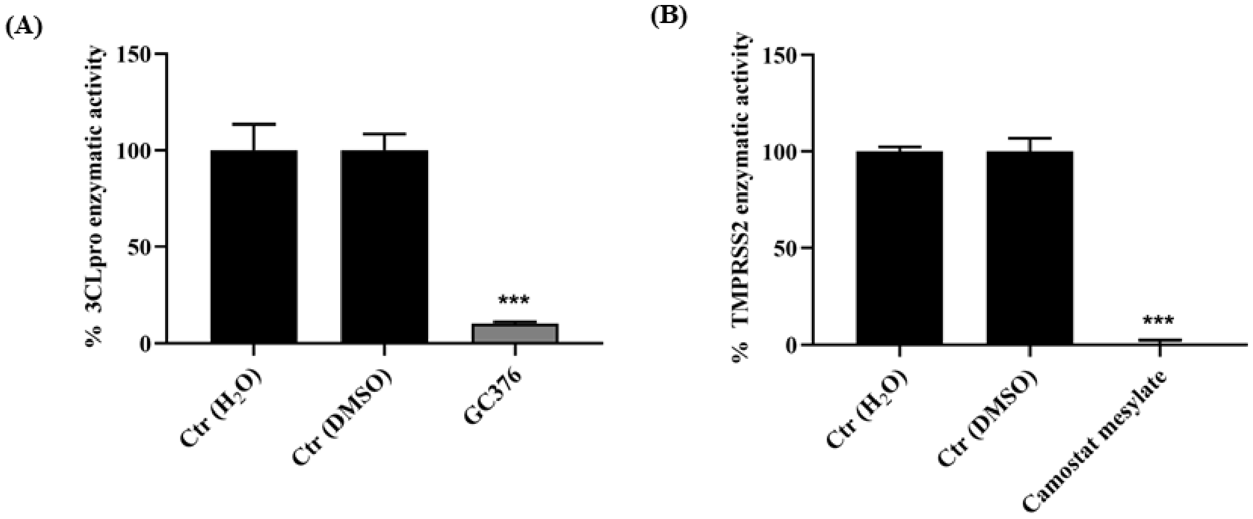


**Figure S2.** Quality control of SARS-CoV-2 3-chymotrypsin like protease (3CLpro) and human transmembrane protease serine 2 (TMPRSS2) enzymatic activity. Tannic acid (TA) was dissolved in water; 1,3,6-tri-O-galloyl-β-D-glucose (TGG) and corilagin were dissolved in DMSO. Both water and DMSO, with 3CLpro or TMPRSS2 and fluorescent substrate, were used as positive controls with no enzyme inhibition as indicated. (**A**) 100 μM GC367 served as the standard inhibitor of 3CLpro enzymatic activity. (**B**) 10 μM camostat mesylate served as the standard inhibitor of TMPRSS2 enzymatic activity. The fluorescence units were converted to a percentage of enzymatic activity considering the positive control wells as 100% activity. Blank values were subtracted from all the readings before calculating the percent activity. Results are expressed as mean ± SD ($n$ = 3). Statistical analysis was performed using the one-way ANOVA followed by the Tukey post hoc test with ***$p < 0.001$ compared to positive control wells.

**Table S1.** Kinetics of TA/protein association and dissociation as measured by the SPR method.

| Protein | $K_a$ ($M^{-1}$ $s^{-1}$) | $K_d$ ($s^{-1}$) | $K_D$ (M) |
|---|---|---|---|
| **RBD (N501Y)** | $2.541 \times 10^{3}$ | $1.066 \times 10^{-4}$ | $4.198 \times 10^{-8}$ |
| **TMPRSS2** | $1.289 \times 10^{4}$ | $1.506 \times 10^{-4}$ | $1.168 \times 10^{-8}$ |
| **3CLpro** | $3.060 \times 10^{3}$ | $1.759 \times 10^{-4}$ | $5.747 \times 10^{-8}$ |

Binding kinetics of TA over the immobilized recombinant protein sensor chip were evaluated in phosphate buffered saline (PBS) + 0.05% Tween buffer with increasing polyphenol concentrations (1 to 80 μM) at a flow rate of 20 μL/min. Association time was set at 300 s and dissociation time was extended up to 1,200 s. The sensor chip surface was regenerated by injecting 10 μL of 50 mM NaOH solution at a flow rate of 20 μL/min. Binding sensograms were obtained by subtracting the reference flow cell (without protein). Experiments were performed at least in duplicate and data analysis was performed using the BIA evaluation software package (GE Healthcare) and fit to a one-site (1:1 molecular ratio) Langmuir binding model.

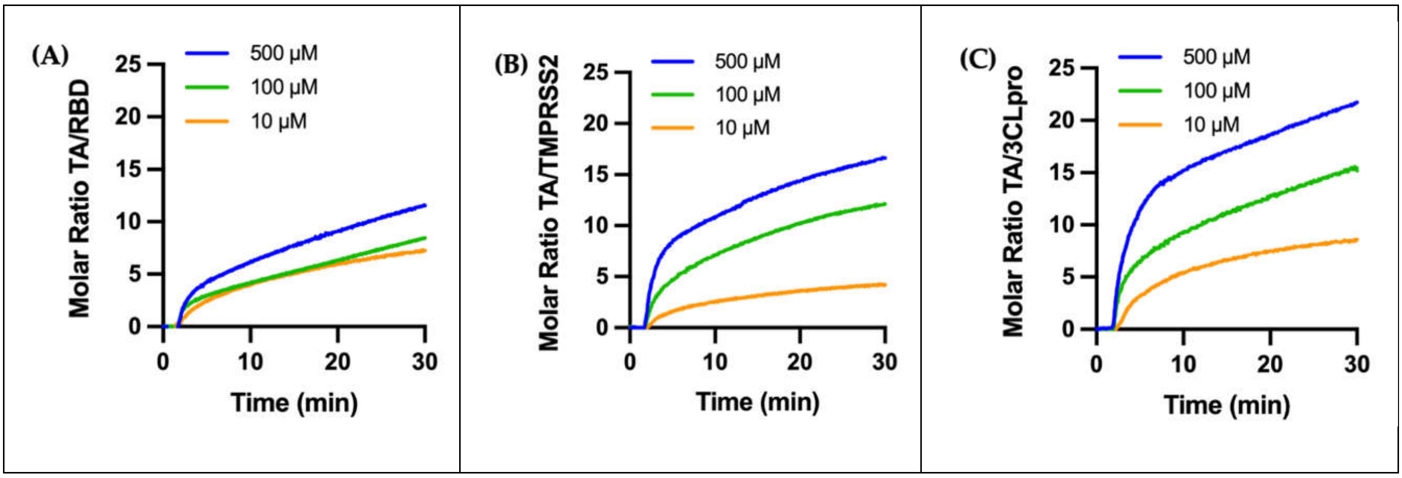


**Figure S3.** Kinetics of TA adsorption to protein-coated surfaces as measured by quartz crystal microbalance with dissipation monitoring (QCMD), expressed as the dimensionless molar ratio of adsorbed TA from solution to adsorbed protein, for: (**A**) TA/RBD, (**B**), TA/TMPRSS2, and (**C**) TA/3CLpro systems. When particles adsorb to the QCMD sensor, water molecules within (intrinsic) and between particles in the adsorbed layer are also sensed in the frequency shift [63,83]. Here we hypothesize a similar water weight fraction for TA and protein to normalize and estimate the dimensionless molar (TA:protein) ratio. The linear increase in adsorbed mass towards 30 minutes is due to bi- and multilayer adsorption of TA.

A monolayer of adsorbed TA occurs at the intersection between the steepest initial slope of the molar ratio-time curve (due to the adsorption of TA on protein) and the second slope (due to multilayer adsorption of TA on TA). It can be seen from Figure S3 that the amount of adsorbed TA at monolayer coverage increases with TA concentration. A likely explanation is that at low TA concentrations TA adsorbs in a flat configuration, whereas at high concentrations TA adsorbs mainly edge-on and/or as aggregates.

**Table S2.** Hydrogen bonds and other bond types between TA and protein residues. The first column lists the proteins and their best poses. The second column indicates the residues of those proteins involved in the formation of H-bonds with TA, while the third column indicates the donor/acceptor H-bond lengths. The last column shows additional bonds, including amino acids with hydrophobic side chains, which are shown in bold. These bonds are from the 1000 ns molecular dynamics (MD) simulations.

| Protein (Pose) | H-Bond (Residue) | H-Bond length $1^{st}/2^{nd}/3^{rd}$ (Å) | Other residues binding with TA |
|---|---|---|---|
| RBD-N501Y (1) | Phe490 | 2.99 | Ser349, **Tyr351**, **Ala352**, Gln439, Gly447, **Tyr449**, Ans450, **Leu452**, **Leu455, Tyr473**, **Tyr489,** Pro491, **Leu492,** Gln498, Gly504 (X2) |
| | Ser494 | 3.15 | |
| | Gly496 | 3.17 | |
| | Val503 | 3.22 | |
| | Tyr505 | 3.08 | |
| RBD-N501Y (2) | Ala 475 | 2.96 | **Phe456,** Gln474, Gly476, Asn487, Cys488, **Tyr489,Phe490**, Pro491 |
| RBD-N501Y (3) | Gln 474 | 3.30 | **Leu452**, **Tyr473, Tyr489**, **Phe490**, Pro491, Gln493 |
| | Leu 492 | 3.20 | |
| | Ser 494 | 3.18/3.26 | |
| RBD-N501Y (4) | Arg346 | 3.14 | **Phe347**, **Ala348**, Asp442, Ser443, Asn448, Asn450, **Leu452**, **Val483**, Gln484, Asn487, **Tyr489**, Ser494 |
| | Tyr351 | 2.97 | |
| | Leu441 | 2.96/3.03 | |
| | Lys444 | 3.07 | |
| | Val445 | 3.00/3.28 | |
| | Tyr449 | 3.04 | |
| | Gly485 | 2.99/3.28 | |
| | Cys488 | 3.05 | |
| | Phe490 | 3.01/2.67 | |
| | Leu492 | 3.33/3.03 | |
| TMPRSS2 (1) | Ser84 | 2.74/2.89 | **Ala40**, His41, Cys(Cyx)42, Thr86, Lys87, **Tyr159, Val160**, **Leu164,** Gln183, Gly184,Ser186, **Trp206**, |
| | Lys85 | 2.78/2.89 | |
| | Asp90 | 3.04 | |
| | Glu134 | 2.93/2.53 | |
| | Lys135 | 3.23 | |
| | Arg158 | 3.07 | |
| | Lys212 | 2.80 | |
| TMPRSS2 (2) | Tyr82 | 2.85 | Ser84, Lys85, Thr86, Lys87, **Leu164**, **Ile165**, **Trp206**, **Val218** |
| | Asp90 | 2.64 | |
| | Thr166 | 3.01/2.82/3.18 | |
| TMPRSS2 (3) | Lys135 | 3.00/3.13 | Lys85, Thr86, Asp162, **Tyr161Leu164**, Thr166, Ser181, Gly184, Asp185, **Trp206**, Gly207, Ser208, Gly209, **Ala211, Ala213**, Arg215 |
| | Val160 | 2.88 | |
| | Ser186 | 3.12 | |
| TMPRSS2 (4) | Val25 | 3.18 | **Val20**, Gln21, Asn22, **Val23,** His41, Cyx42, Lys45, Pro46, His52, **Trp53**, **Tyr67**, **Ala131,** Gly136, Lys137, Thr138, Asp185 |
| | Gly68 | 2.77 | |
| | Gln72 | 3.04/3.06/3.21 | |
| | Lys135 | 2.90 | |
| | Gly184 | 2.95 | |
| 3CLpro (1) | Cys145 | 3.23 | Gly23,Thr25, His41,**Val42**, Cys44,Thr45, Ser46, **Met49**, **Leu50**, **Met165,** Glu166,**Leu167,** Pro168, Asp187, Arg188, Gln189, Thr190 |
| | Cys22 | 2.59/2.71 | |
| | Ala191 | 3.01 | |
| | Gln192 | 2.96 | |

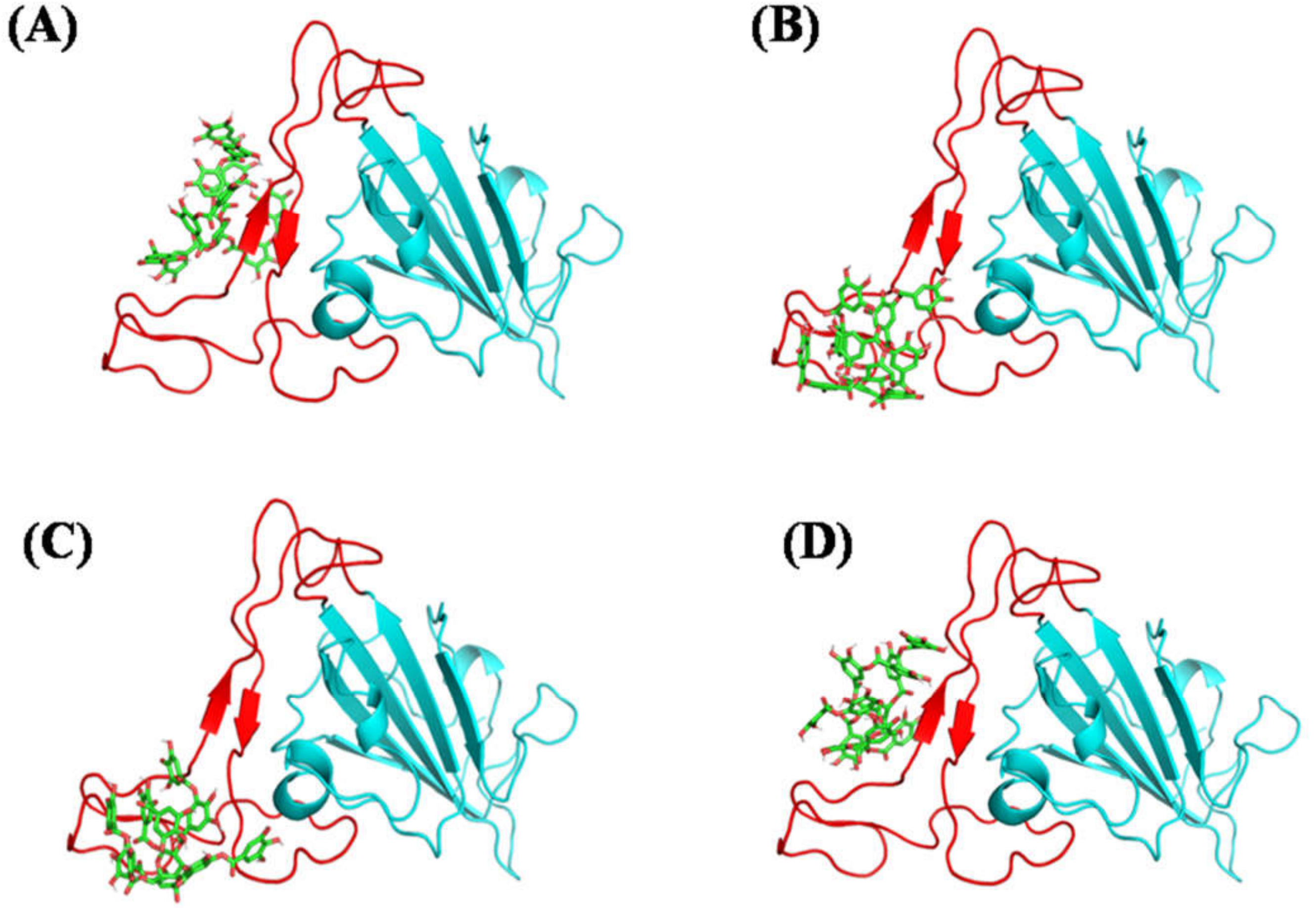


**Figure S4.** Molecular structures of TA/RBD (N501Y) complexes after docking (poses 1–4) and VINA binding affinities: (**A**) pose 1,-6.8; (**B**) pose 2, -6.7; (**C**) pose 3, -6.7; and (**D**) pose 4, -6.6 kcal/mol. The figures were generated using PyMOL v2.5.0 [108].

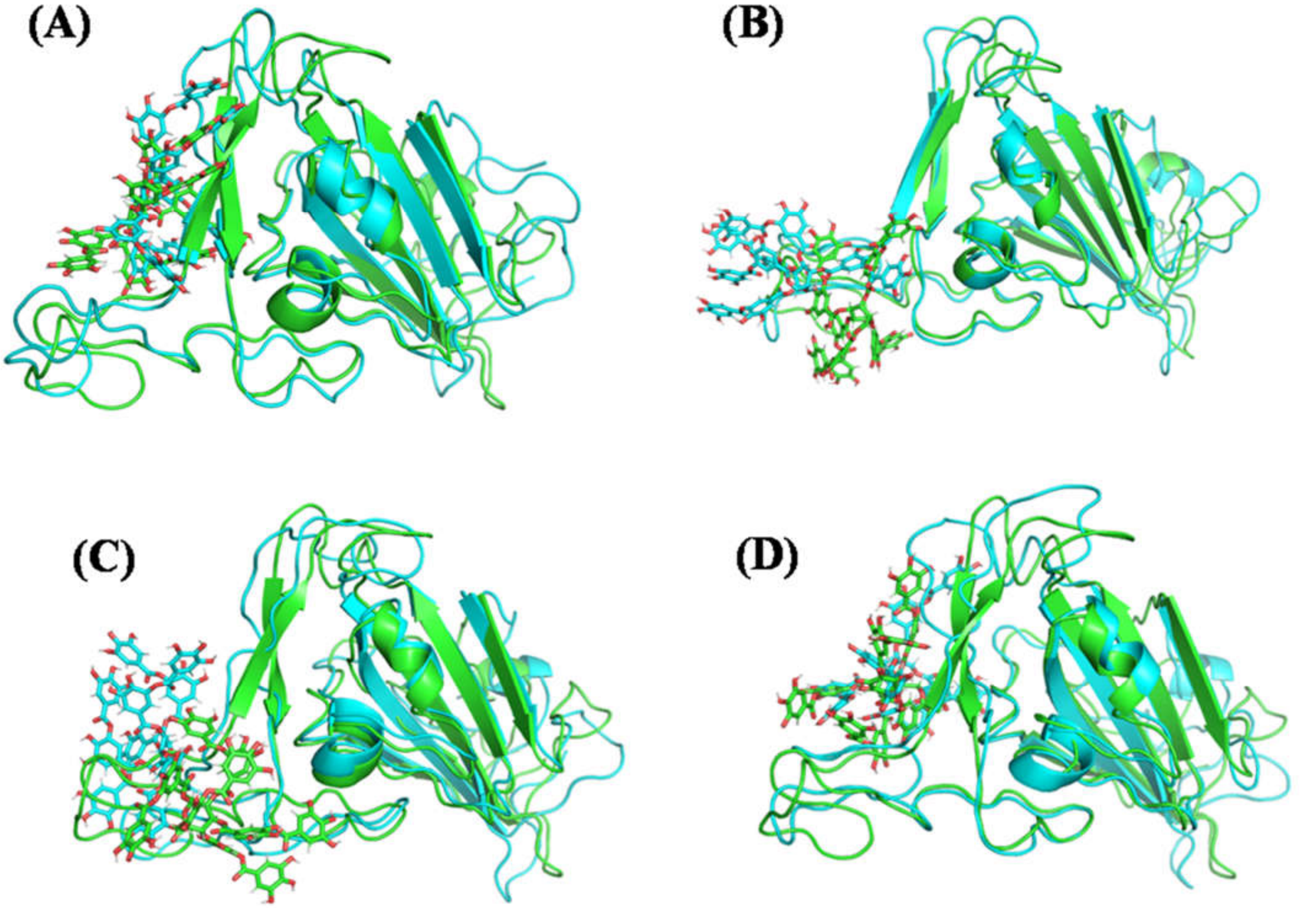


**Figure S5.** Molecular structures of TA/RBD (N501Y) complexes before (green) and after (turquoise) 1000 ns MD simulations: (**A**) pose 1; (**B**) pose 2; (**C**) pose 3; and (**D**) pose 4. The figures were generated using PyMOL v2.5.0 [108].

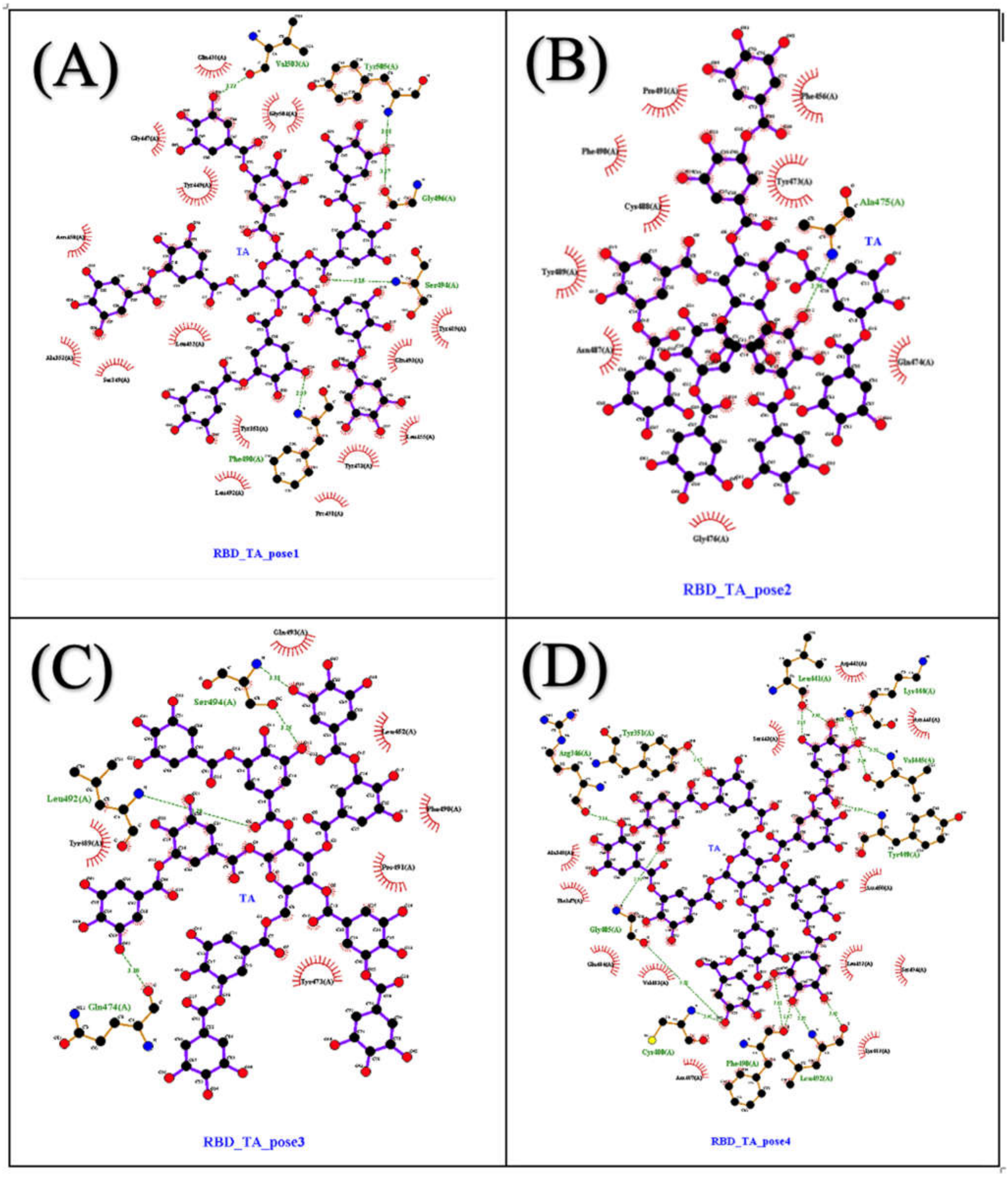


**Figure S6.** Tannic acid interaction maps: the interaction maps of TA/RBD (N501Y) complexes for the center of the four biggest clusters (**poses 1 (A), 2 (B), 3 (C),** and **4 (D)**) computed on the convergence interval using the protein backbone atoms and ligand non-hydrogen atoms. The other contacts, defined by a distance smaller than 0.40 nm, between the ligand and the protein are shown as red arcs. H-bonds and their donor/acceptor distances are shown in green. The interaction map was generated using LigPlot [51,52].

**(A)** **(B)**

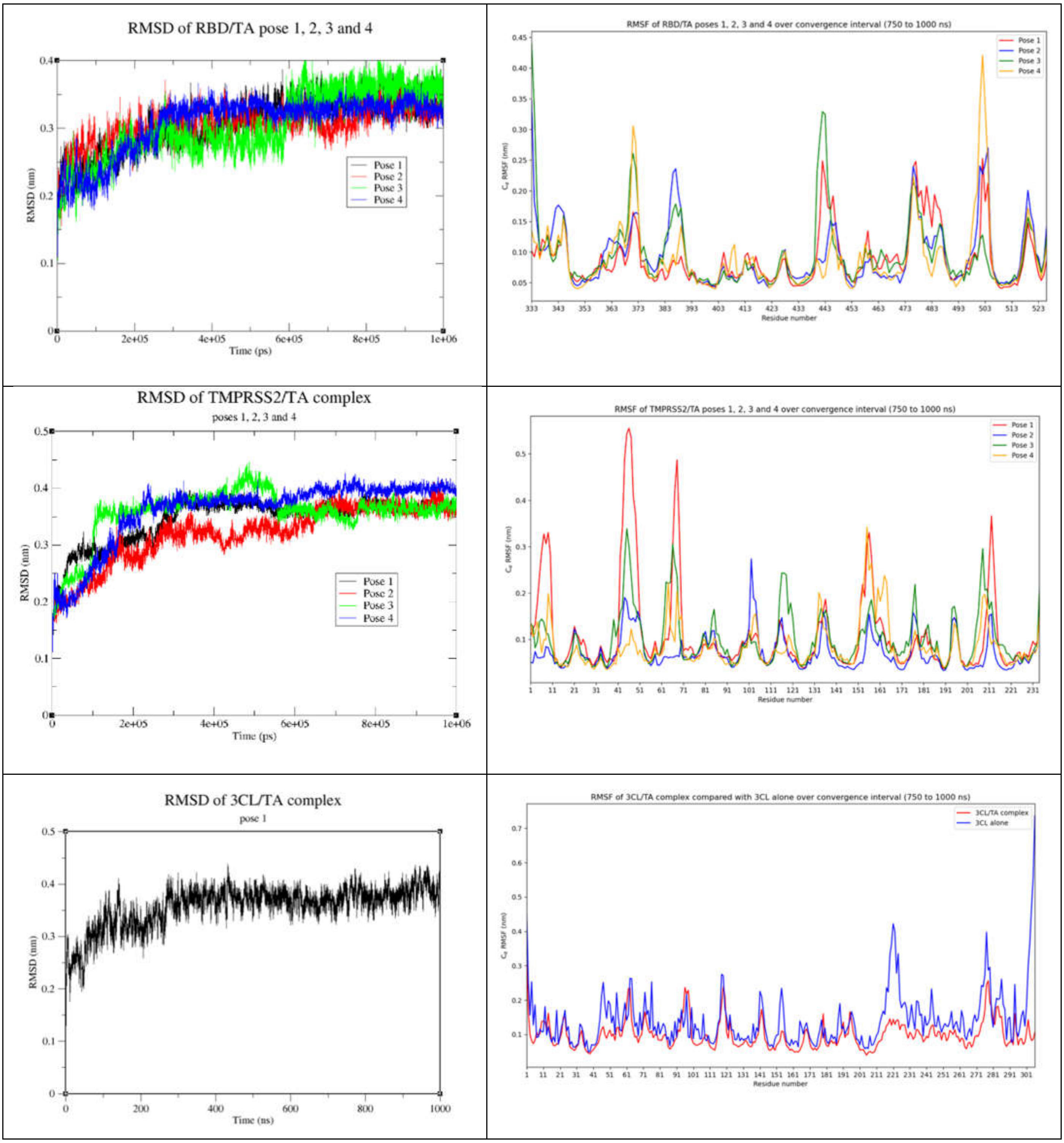


**Figure S7.** Convergence of the TA/protein complexes over the MD trajectory of TA/RBD (N501Y) (poses 1–4), TA/TMPRSS2 (poses 1–4), and TA/3CLpro (pose 1). (**A**) Root mean square deviation (RMSD) on the backbone atoms (N, Cα, C and O) from the protein structures as a function of time (1000 ns); and (**B**) the root mean square fluctuations (RMSF) per residue for the 750–1000 ns interval.

**(A)** **(B)**

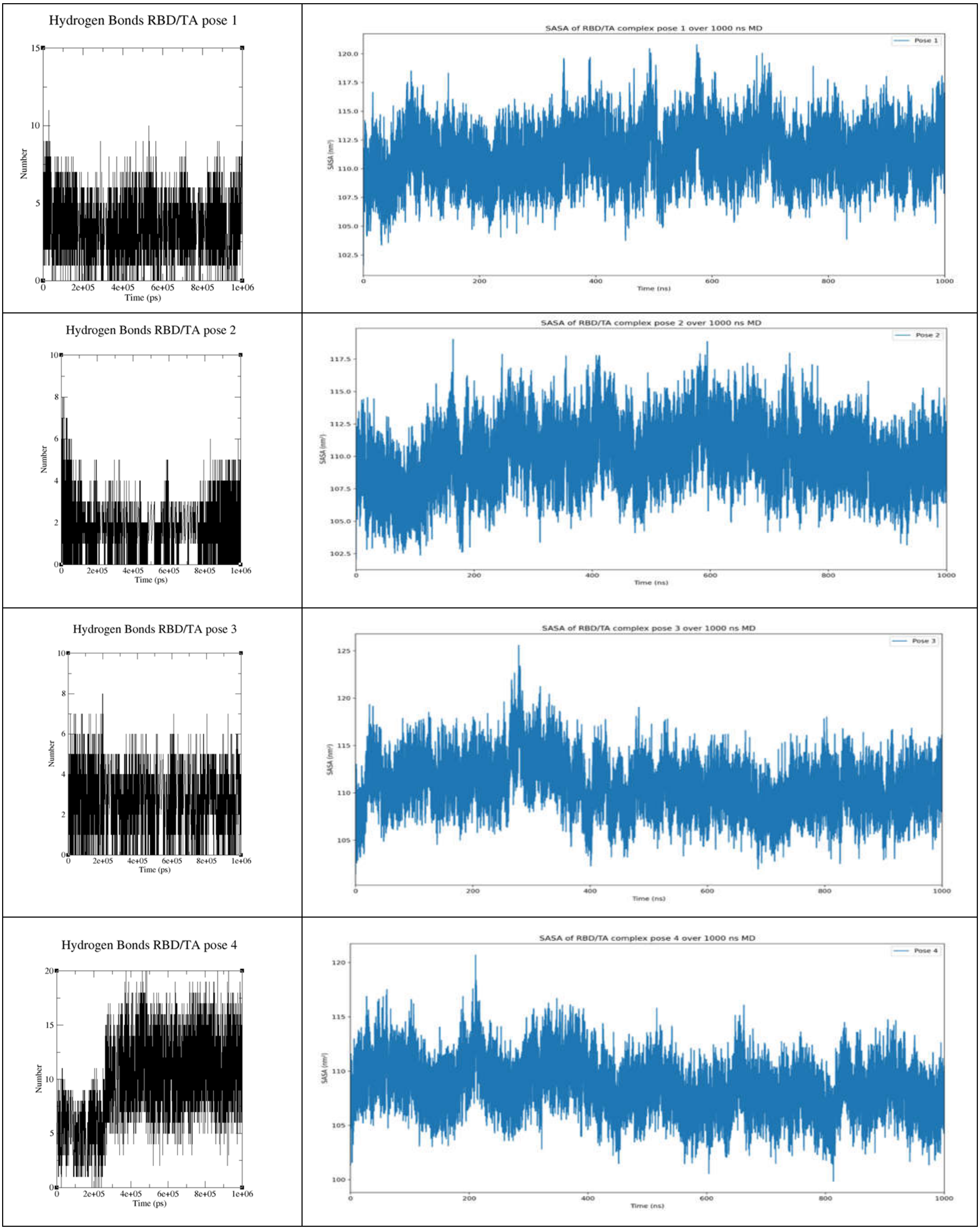


**Figure S8.** (**A**) Number of H-bonds of TA/RBD (N501Y) complexes (poses 1 to 4); and (**B**) the solvent accessible surface area (SASA); both over 1000 ns MD simulations.

**(A)** **(B)**

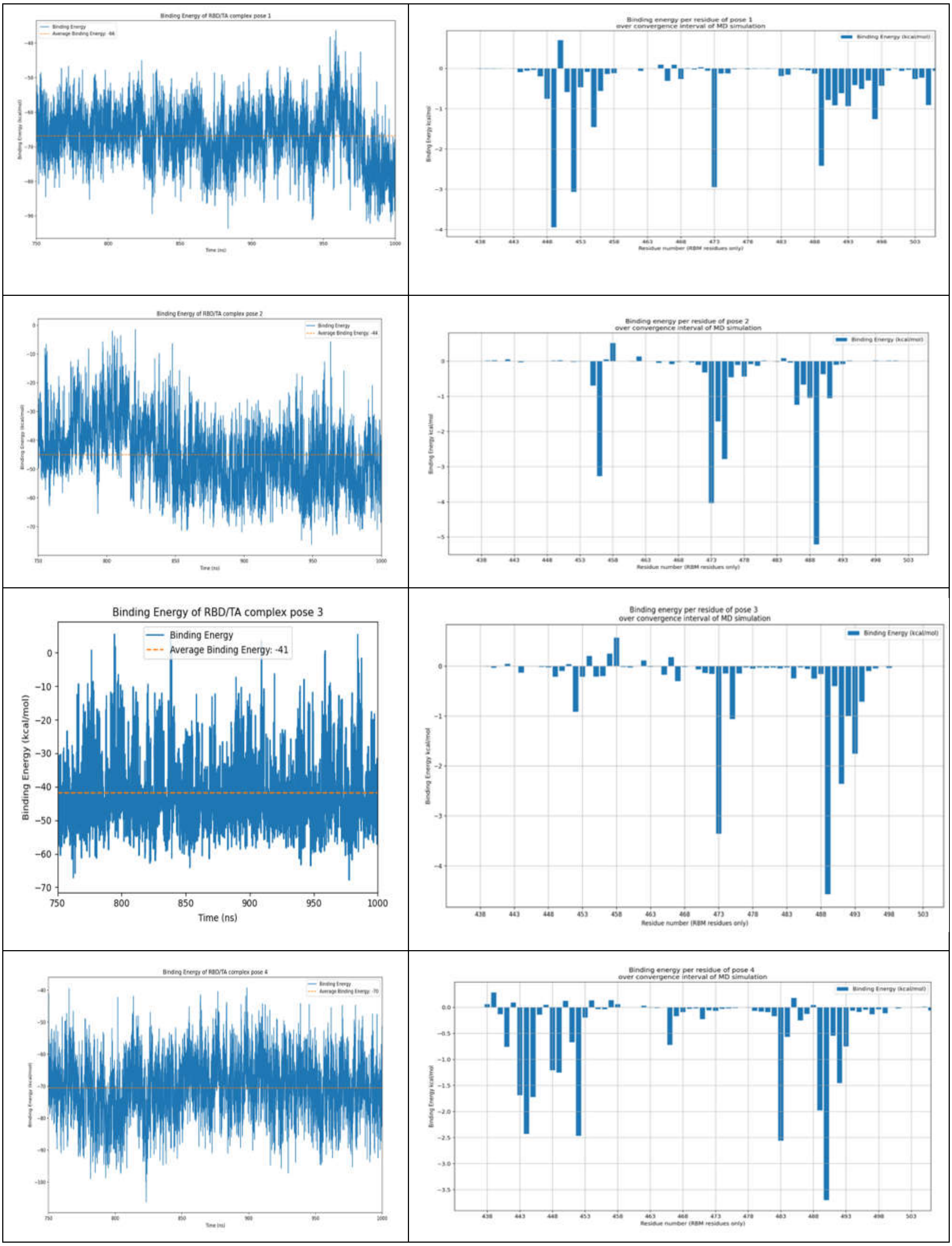


**Figure S9.** (**A**) Molecular Mechanics Poisson-Boltzmann Surface Area (MMPBSA) binding free energy of TA/RBD (N501Y) complexes over 1000 ns (poses 1 to 4); and (**B**) the binding free energy per residue over the convergence interval (750–1000 ns) of MD simulations.

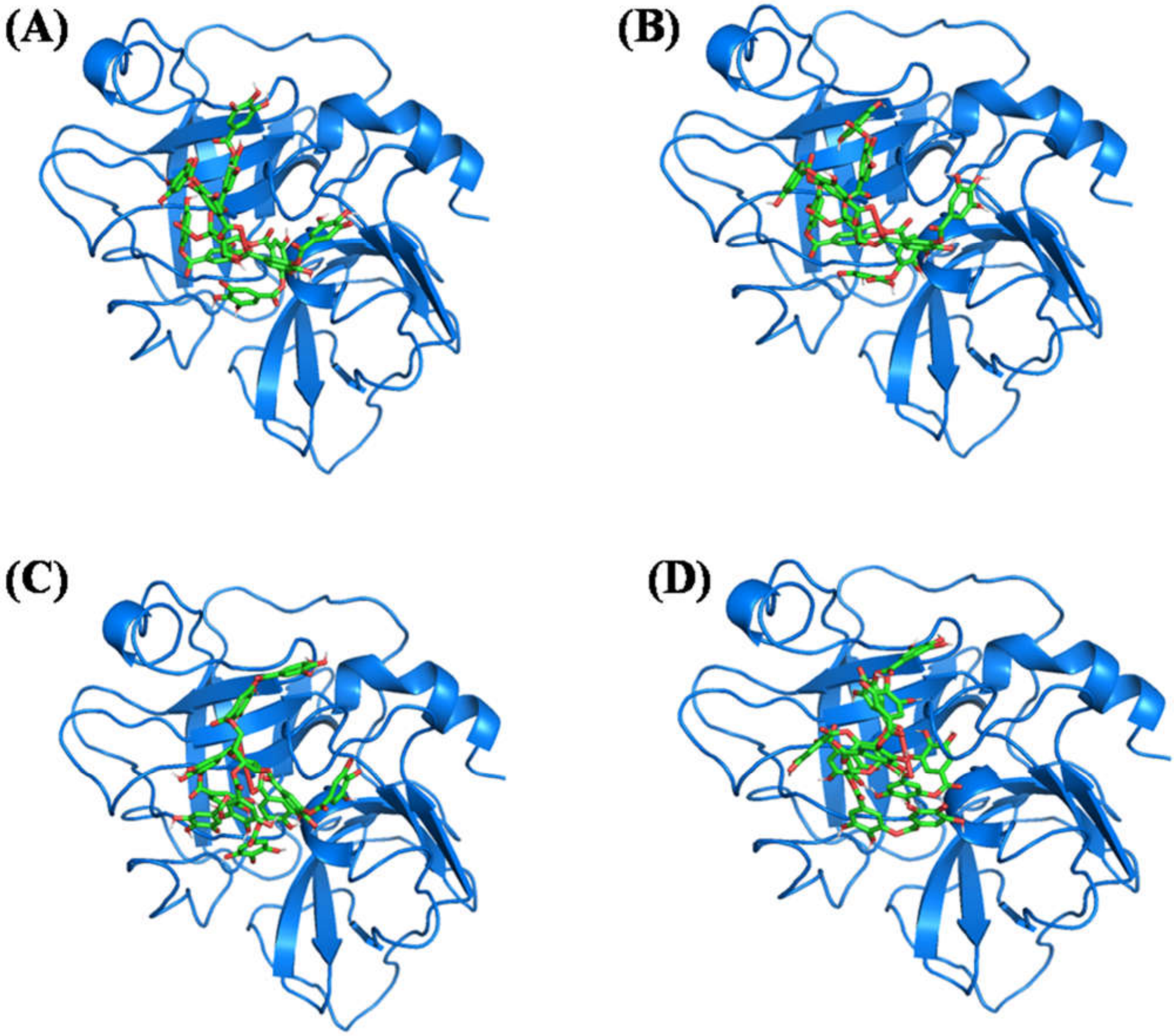


**Figure S10.** Molecular structures of TA/TMPRSS2 complexes after docking (poses 1–4) and VINA binding affinities : (**A**) pose 1, -2.2; (**B**) pose 2, -1.6; (**C**) pose 3, +0.9; and (**D**) pose 4, +1.5 kcal/mol. The figures were generated using PyMOL v2.5.0 [108].

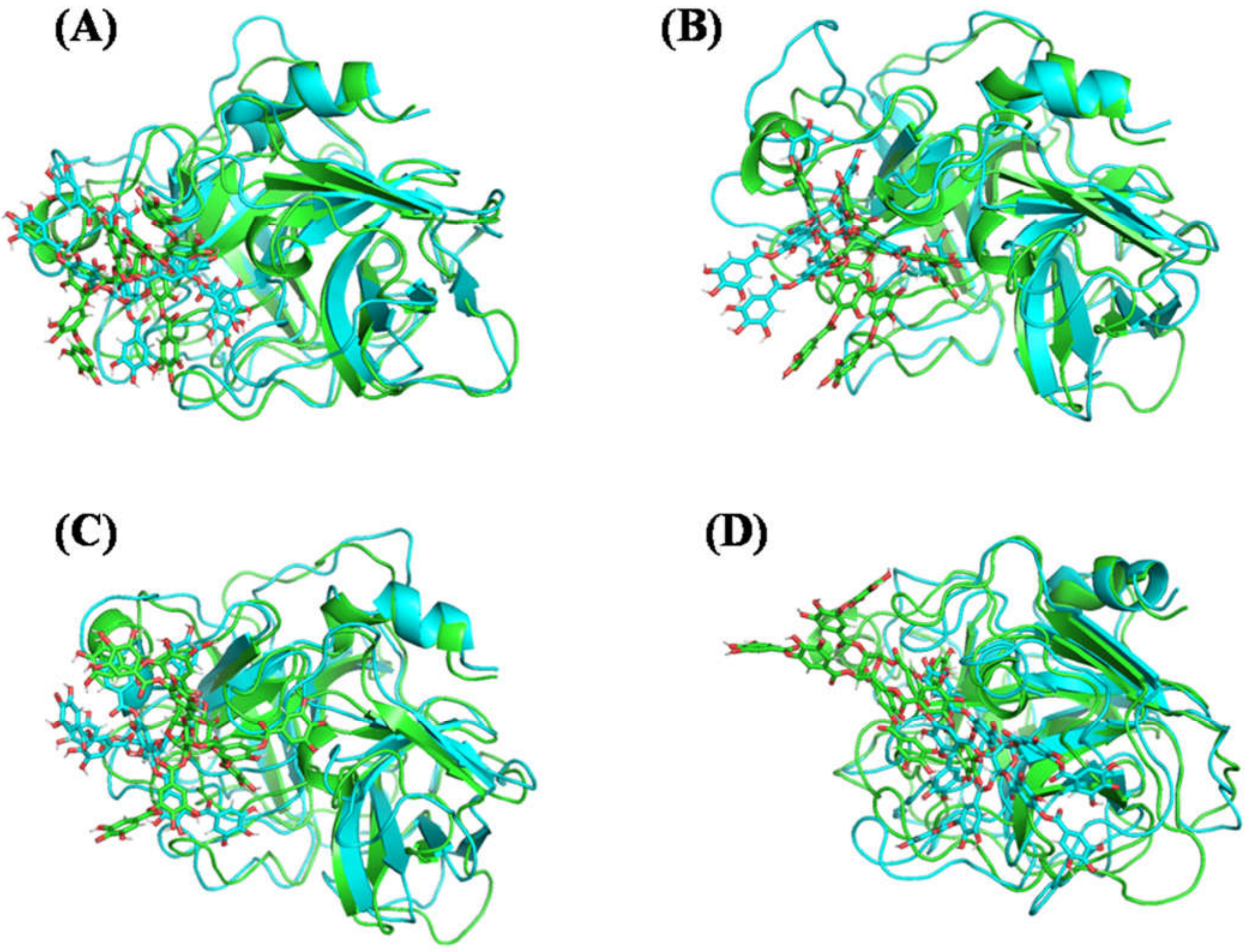


**Figure S11.** Molecular structures of TA/TMPRSS2 complexes before (green) and after (turquoise) 1000 ns MD simulations: (**A**) pose 1; (**B**) pose 2; (**C**) pose 3; and (**D**) pose 4. The figures were generated using PyMOL v2.5.0 [108].

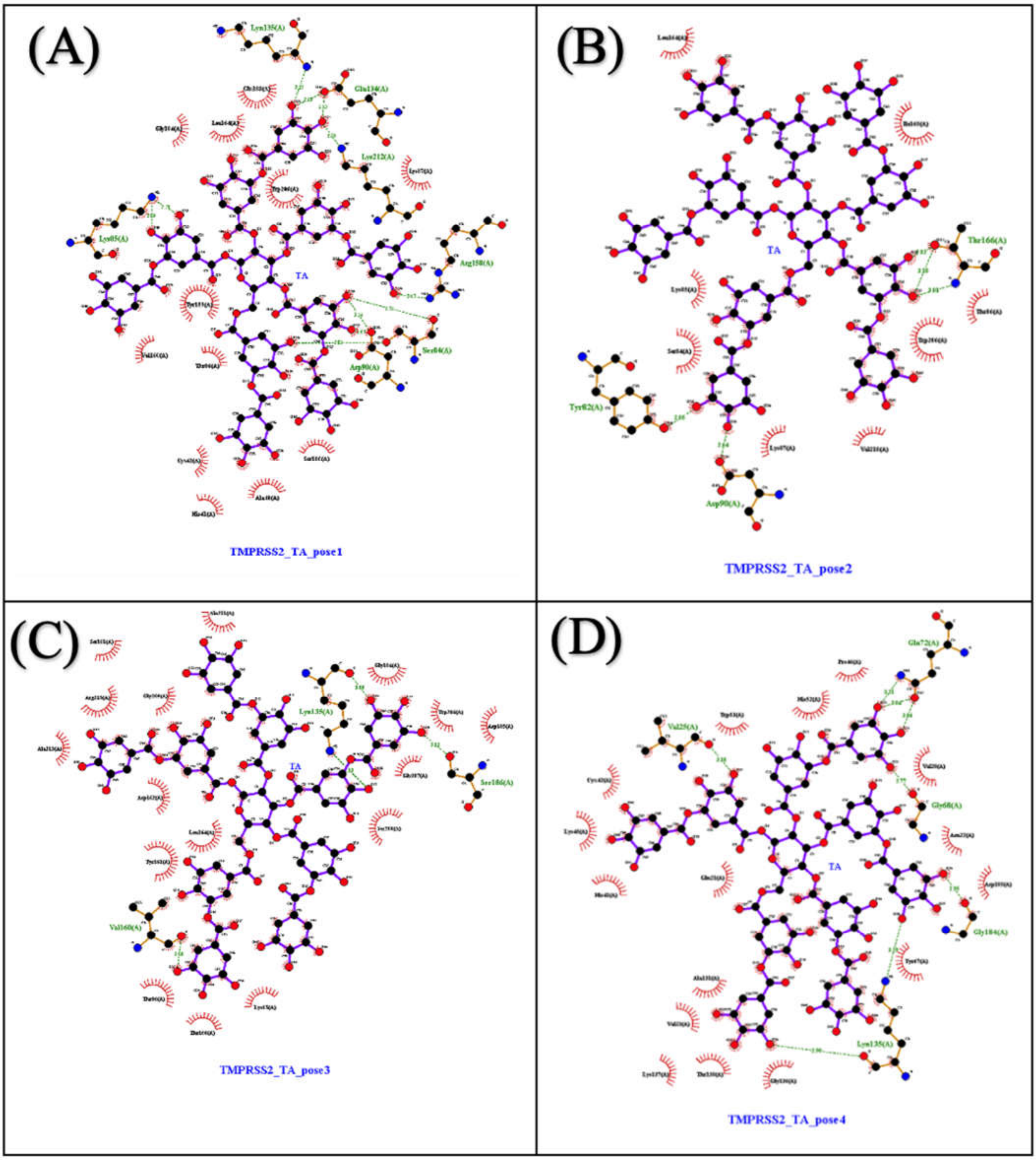


**Figure S12.** Tannic acid interaction maps: the interaction maps of TA/TMPRSS2 complexes for the center of the biggest four clusters (**poses 1 (A), 2 (B), 3 (C),** and **4 (D)**) computed on the convergence interval using the protein backbone atoms and ligand non-hydrogen atoms. The other contacts, defined by a distance smaller than 0.40 nm between the ligand and the protein are shown as red arcs. H-bonds and their donor/acceptor distances are shown in green. The interaction map was generated using LigPlot [51,52].

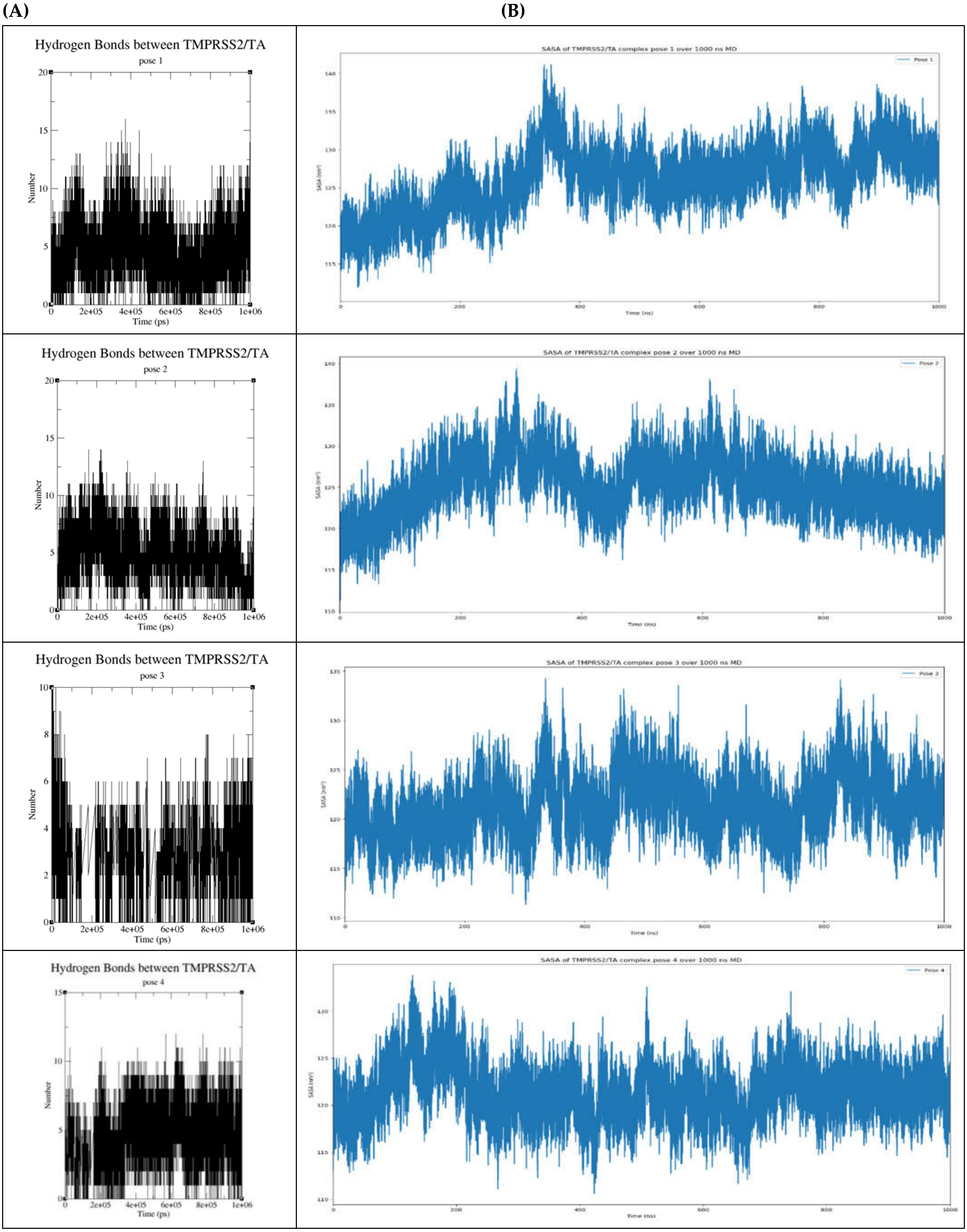


**Figure S13**. (**A**) Number of H-bonds of TA/TMPRSS2 complexes (poses 1 to 4); and (**B**) solvent accessible surface area (SASA); both over 1000 ns MD simulations.

**(A)** **(B)**

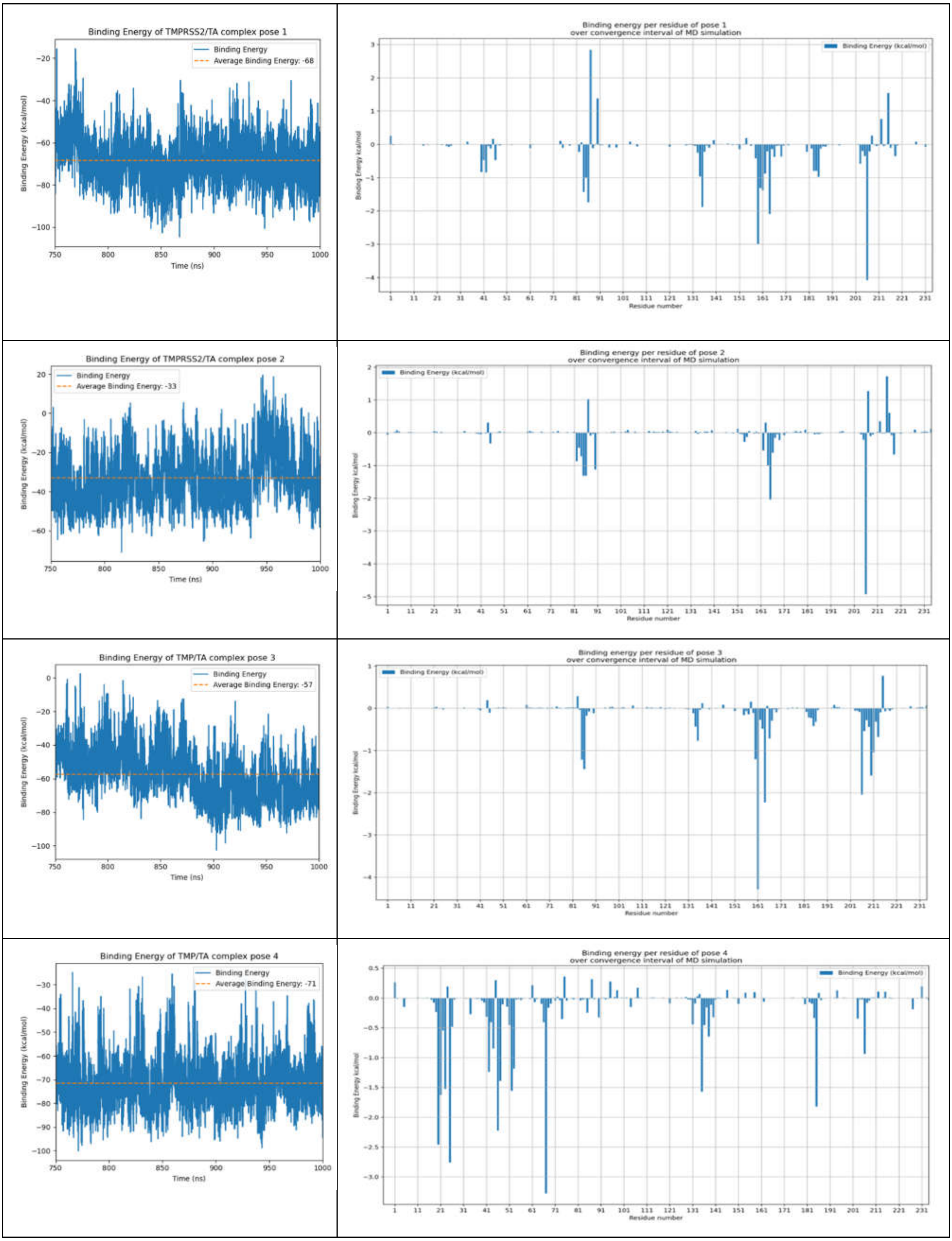


**Figure S14.** (**A**) MMPBSA binding free energy of TA/TMPRSS2 complexes over 1000 ns (poses 1 to 4); and (**B**) the binding free energy per residue over the convergence interval (750–1000 ns) of MD simulations.

**(A)** **(B)**

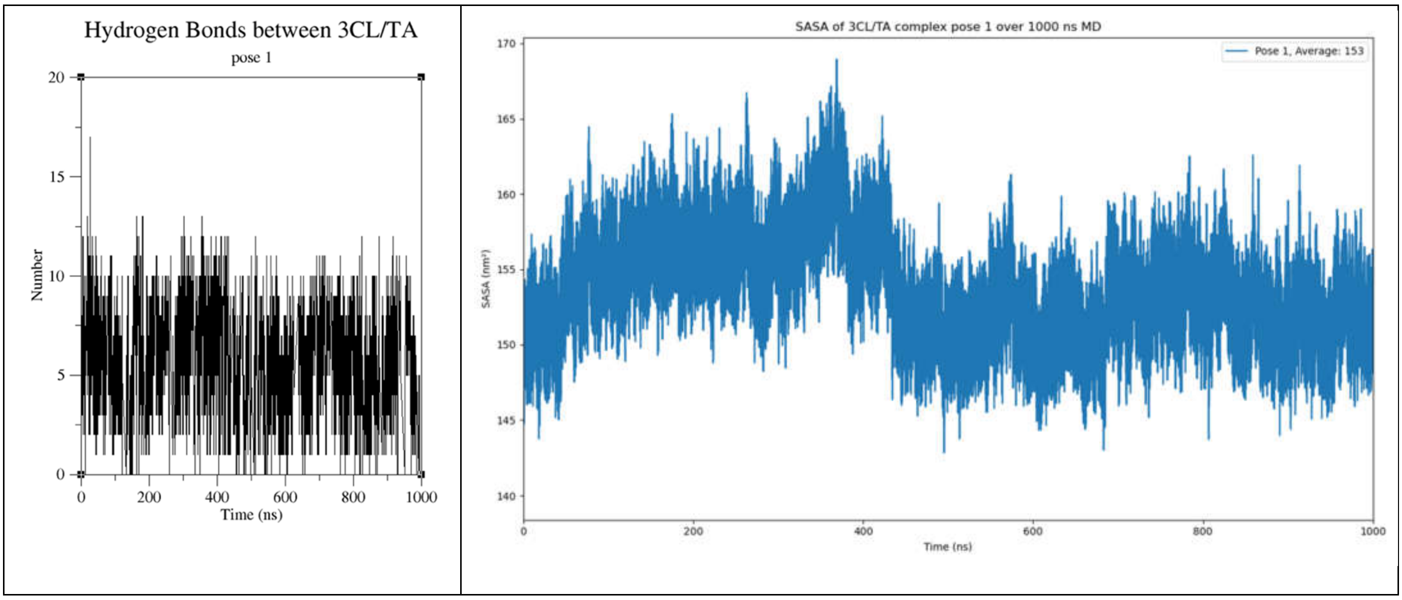


**Figure S15.** (**A**) Number of H-bonds of the TA/3CLpro complex (pose 1); and (**B**) the solvent accessible surface area (SASA); both over 1000 ns MD simulations.

**(A)** **(B)**

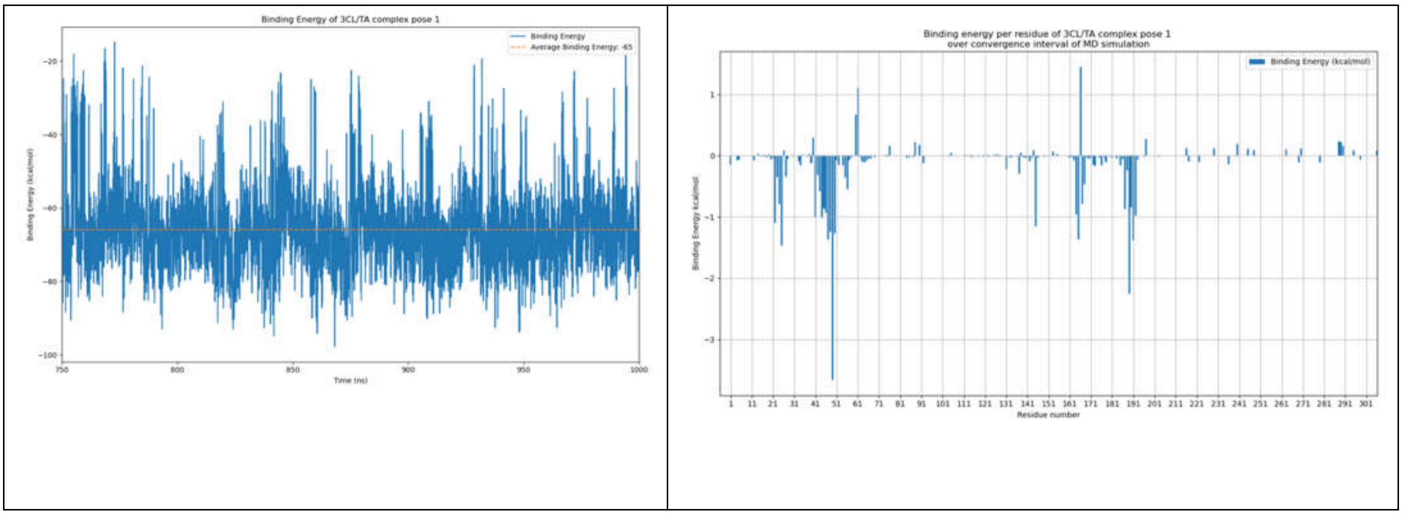


**Figure S16.** (**A**) MMPBSA binding free energy of the TA/3CLpro complex over 1000 ns (pose 1); and (**B**) the binding free energy per residue over the convergence interval (750–1000 ns) of MD simulations.

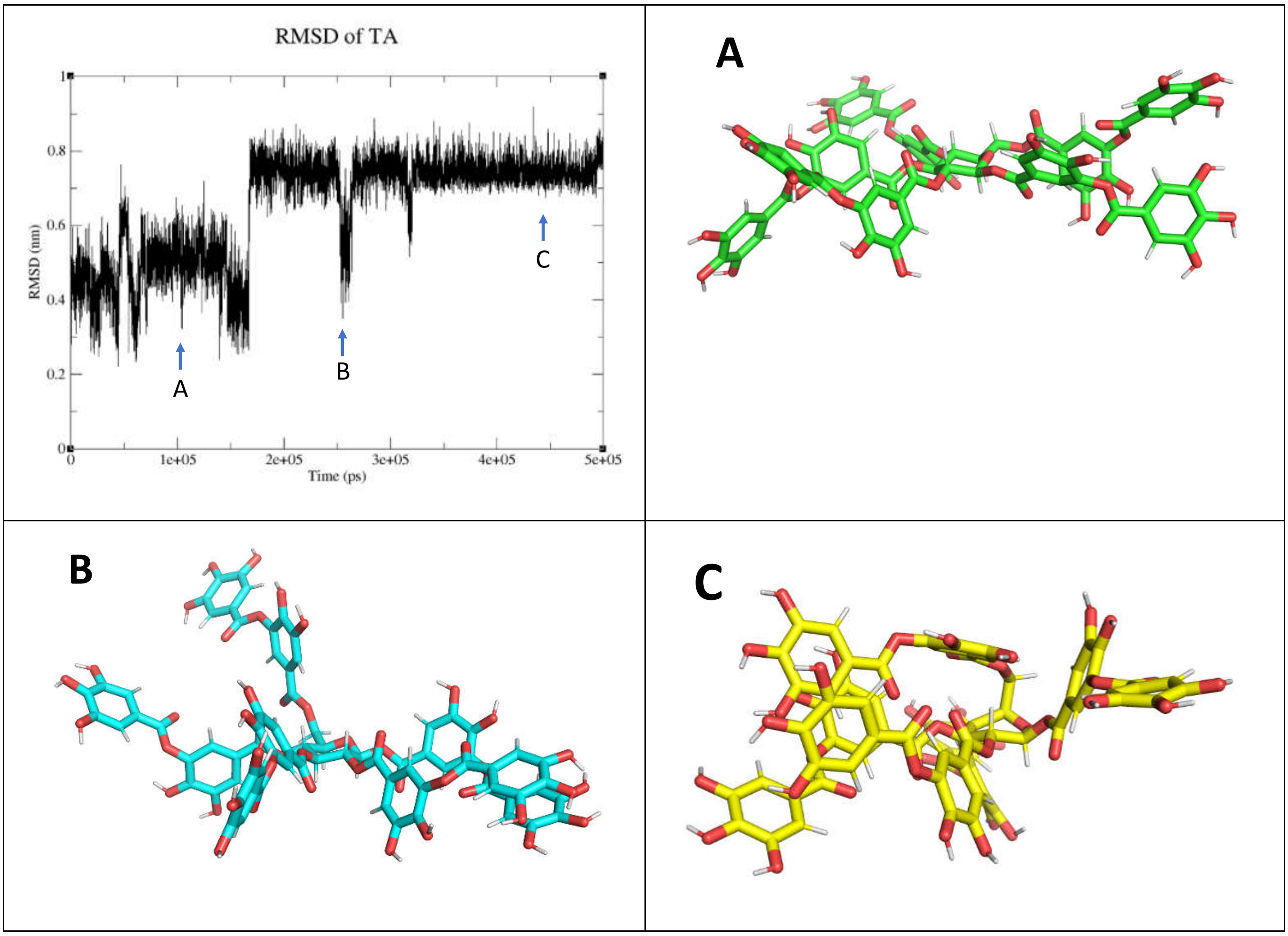


**Figure S17.** (top left) Major RMSD conformational changes of the sugar ring of TA over a 500 ns MD simulation: (A) chair at 100 ns, (B) chair at 260 ns and (C) skew-boat at 450 ns (calculated with GROMACS v2021.2).

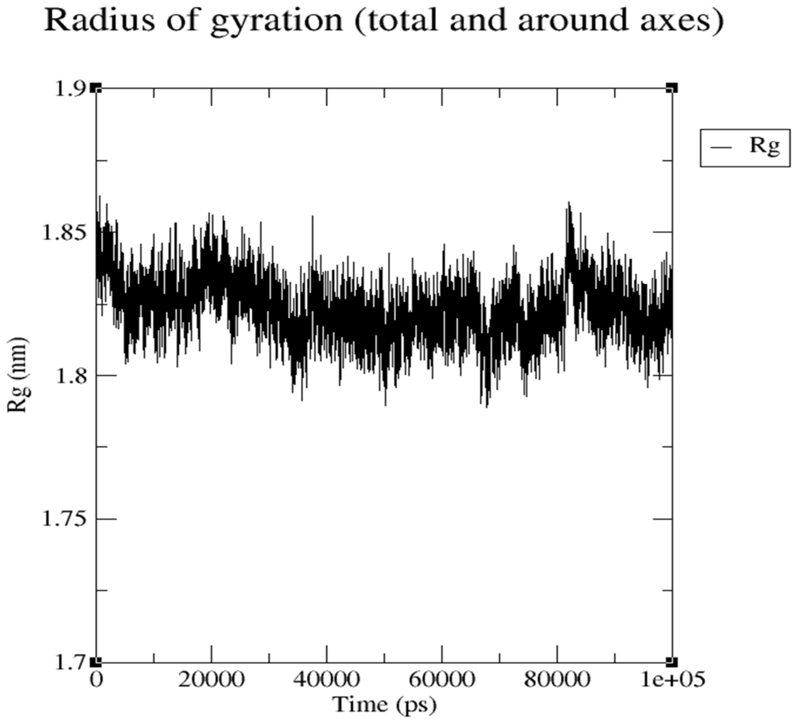


**Figure S18.** Radius of gyration of RBD (N501Y) protein of approximately 1.825 nm (calculated with GROMACS v2021.2).

**Table S3.** Sequences for recombinant RBD (N501Y), human TMPRSS2 and 3CLpro proteins used for experimental and theoretical (in-silico) assessment of TA/protein complexes.

| Methods | RBD (N501Y) | TMPRSS2 | 3CLpro |
|---|---|---|---|
| **Experimental (Enzymatic assays/SPR/QCMD)** | 319–541 | 106–492 | 1–306 |
| **In-silico** | 333–526 | 1–234 | 1–306 |
